# Sentinel nurseries to assess the phytosanitary risks from insect pests on importations of live plants

**DOI:** 10.1038/s41598-018-29551-y

**Published:** 2018-07-25

**Authors:** Marc Kenis, Hongmei Li, Jian-ting Fan, Beatrice Courtial, Marie-Anne Auger-Rozenberg, Annie Yart, René Eschen, Alain Roques

**Affiliations:** 1 CABI, 2800 Delémont, Switzerland; 2 MoA-CABI Joint Laboratory for Bio-safety, Institute of Plant Protection, Chinese Academy of Agricultural Sciences, Beijing, 100193 China; 3 School of Forestry and Bio-technology, Zhejiang Agriculture and Forestry University, Lin’an, China; 4 INRA UR 633 Zoologie Forestière, 45075 Orléans, France

## Abstract

Ornamental plants represent a major pathway of invasion for alien pests worldwide. Commodity risk analyses are carried out to assess the risk posed by a new trade in a commodity, but they are restricted by our limited knowledge of the pests carried by traded plants. We used the sentinel nursery technique to identify insects attacking woody plants imported into Europe. We established two sentinel nurseries in China, with five traded Asian plants. These nurseries were monitored for two years to obtain lists of insects that can be expected on these commodities. These records were compared with those obtained from literature surveys, which are usually the sources of information available to pest risk assessors. At each site, 105 insect species and host associations were found on sentinel plants and 90% of these associations had not been found in a previous literature survey of insect pests of the five plants. Nearly 80% of these associations were not found in an *a posteriori* literature survey. An assessment classified 9%, 7% and 84% of the insect records as presenting a high, moderate and low likelihood of introduction, respectively. These results show the benefit of sentinel nurseries to identify potential infestation of plant commodity imports.

## Introduction

The trade in live plants, in particular ornamentals, is considered to be the main pathway of introduction for invasive alien plant pests^1–4^. Every year, the number of traded live plants and plant species increases, representing new threats for the environment and economy worldwide^2,5^. Europe is particularly vulnerable because of its rather permissive legislation regarding the importation of live plants^3,6^, but no continent is spared. Examples of devastating plant pests that most likely travelled with live plants in the last two decades are the citrus longhorn beetle (*Anoplophora chinensis*), the box tree moth (*Cydalima perspectalis*), the light brown apple moth (*Epiphyas postvittana*), box blight (*Calonectria pseudonaviculata*), ash dieback (*Hymenoscyphus fraxineus*), and various *Phytophthora* species^2,7,8^.

Pest risk analyses (PRAs) are carried out to assess the risk posed by emerging or recently introduced plant pests^9,10^. However, PRA is restricted by our limited knowledge of the pests that imported plants may vector^11,12^. Indeed, most pests and pathogens recently introduced on live plants had not been previously identified as quarantine pests and, thus, had not been subject to specific monitoring by inspection services at ports of entry^13^. For this reason, commodity risk analyses (CRA), also called import or pathway risk analyses, are increasingly being employed to assess the risk associated with the import of a particular commodity (e.g. a plant species) from a particular region^14,15^. However, CRA are usually based on the identification of the most likely and known pests associated with a commodity on which a PRA will be made. The list of the selected pests is based on literature searches rather than on the actual pests that are commonly carried on the assessed commodity. Thus, a CRA may find that a commodity is not a phytosanitary risk simply because of incomplete information available in the literature.

The sentinel plant method is increasingly advocated as a strategy to identify plant pests in a particular region before they are introduced^16–18^. In a recent experiment named “sentinel plantation”, Roques *et al*.^19^ and Vettraino *et al*.^20^ planted European tree species in China to assess which plant pest could become a threat to these species if introduced accidentally to Europe. They found many pest species on the sentinel plantations that would never have been considered as potential threats without this experiment. Similarly, Tomoshevich *et al*.^21^ surveyed European woody plants in Siberian arboreta to identify potential pathogens that could become threats if they were introduced in Europe.

Sentinel plants, however, could also be used to support CRA. In another approach that we name “sentinel nurseries”, plants that are newly or increasingly traded across continents could be established in the region of export, without phytosanitary treatment, to assess which pests could potentially be transported by these commodities, and compare the information gathered with that of a literature review on the known pests on that commodity or in the exporting country. Furthermore, knowing which pests are associated with particular plant commodities would allow the importing countries to alert inspectors on the possible presence of specific pests and their symptoms, and to develop new surveillance and detection techniques accordingly. Finally, it would also allow the exporting countries to better identify specific pests on which to focus detection and phytosanitary treatments at the export stage.

At two sites in Northern China (Beijing) and Eastern China (Fuyang), five ornamental woody plant species commonly imported from China to Europe (*Acer palmatum* Thunb.*, Ilex cornuta* Lindl.*, Buxus microphylla* Siebold & Zucc*., Fraxinus chinensis* Roxb. and *Zelkovia schneideriana* Handel-Mazzetti) were planted to obtain lists of potential pests that can be expected on these exported commodities and compare them with known pests from the literature. These experiments were done in China because this country is an increasing important supplier of plants for planting to the European plant trade and many new invasive plant pests in Europe have arrived from East Asia in the recent years^22,23^. However, as a proof of concept the study could have been carried out in any exporting country. Although the aim of this study was to survey for insect pests and pathogens, only the results obtained for insects are reported here. Results for pathogens have been reported in Vettraino *et al*.^24^.

## Results

Over 40 families of insects were recovered from the five hosts in Fuyang and Beijing, most belonging to Coleoptera, Lepidoptera and Hemiptera (Table 1). A total of 105 combinations of insect morphospecies and hosts were found on sentinel plants at each of the two established nurseries (Supplementary Information, Tables S1, S2). Of these, 49.5% and 39.5% were identified to species level at Beijing and Fuyang respectively. This includes seven species for which molecular analyses found a match of 98–99% with published sequences (noted as “nr” in Supplementary Information, Tables S1, S2). An additional 10.5% and 13.3% were identified to genus level at Beijing and Fuyang, respectively. Only three of the insects identified to species level were found at both sites. Molecular analyses were more efficient to identify Lepidoptera than species from other orders because the former group is significantly more represented in online genetic databases such as BOLD and GenBank^19^. However, 65 species were barcoded and all sequences have been kept for future comparisons (Supplementary Information, Tables S1, S2).

**Table 1:** Number of insect morphospecies (including identified and unidentified species) by order and family found in Fuyang and Beijing on the five tree species in the two years of survey.

Order/Family	Fuyang					Beijing				
	*Acer*	*Buxus*	*Fraxinus*	*Ilex*	*Zelkova*	*Acer*	*Buxus*	*Fraxinus*	*Ilex*	*Zelkova*
Coleoptera										
Attelabidae	—	—	—	—	—	—	—	—	—	1
Bostrichidae	—	—	—	—	—	—	—	—	—	1
Buprestidae	—	—	—	—	—	—	—	—	—	1
Cerambycidae	—	—	1	1	3	—	—	1	—	2
Cetoniidae	—	—	—	—	—	—	—	1	—	
Chrysomelidae	1	2	4	4	8	3	1	2	—	4
Coccinellidae	—	—	—	—	—	1	—	1	—	1
Curculionidae	—	—	—	—	1	3	—	1	—	1
Elateridae	1	—	—	—	—	—	—	—	—	—
Melolonthidae	1	—	2	—	3	1	—	1	—	1
Rutelidae	2	—	1	—	3	1	—	—	—	2
Hemiptera										
Alydidae	—	—	—	—	—	—	—	1	—	
Aphidae	—	—	—	—	1	—	1	2	1	1
Berytidae	—	—	—	—		1	1	1	—	1
Cicadellidae	—	—	2	—	1	1	1		—	1
Cicadidae	—	—	—	—	—	—	—	2	—	—
Coccidae	—	—	—	1	—	—	—	—	—	—
Coreidae	—	—	—		1	—	—	—	—	—
Derbidae	—	—	—	—	—	1	—	1	—	1
Dictyopharidae	—	—	—	—	—		—	1	—	
Fulgoridae	—	—	—	—	—		—	1	—	
Lygaeidae	—	—	2	—	—	2	1		—	2
Membracidae	—	—	—	—	—	1	1		—	1
Pentatomidae	1	2	2	1	4	2	1	3	—	3
Hymenoptera										
Tenthredinidae		1	2		2	—	—	—	—	—
Lepidoptera										
Arctiidae	—	—	—	—	1	1	—	1	—	1
Crambidae	—	1	—	—	—	—	1	—	—	—
Cossidae	—	—	1	—	—	—	—	—	—	—
Eupterotidae	—	—	1	—	—	—	—	—	—	—
Gelechiidae	—	—	—	—	—	—	—	—	—	1
Geometridae	1	—	3	2	2	3	—	5	—	3
Limacodidae	—	—	2	—	—	2	—	1	2	—
Lymantriidae	2	—	2	1	2	—	—	1	—	2
Noctuidae	2	—	3		3	1	—	1	—	1
Nymphalidae		—	—		1	—	—	—	—	—
Pyralidae	1	—	1	—	—	—	—	1	—	1
Psychidae		—	2	1	—	—	—	—	—	—
Scythrididae	—	—	—	—	—	1	—	—	—	1
Sphingidae	—	—	3	—	—	—	—	1	—	
Tortricidae		—	—	1	—	—	—	1	—	3
Zygaenidae		—	—	1	—	—	—	—	—	—
Unknown	—	—	1	—	—	—	—	—	—	—
Orthoptera										
Unknown		—	1		2	—	—	—	—	—
Unknown	—	—	—	—	—	—	—	1	—	—
Total species	**12**	**6**	**36**	**13**	**38**	**25**	**8**	**32**	**3**	**37**

**Figure 1:**
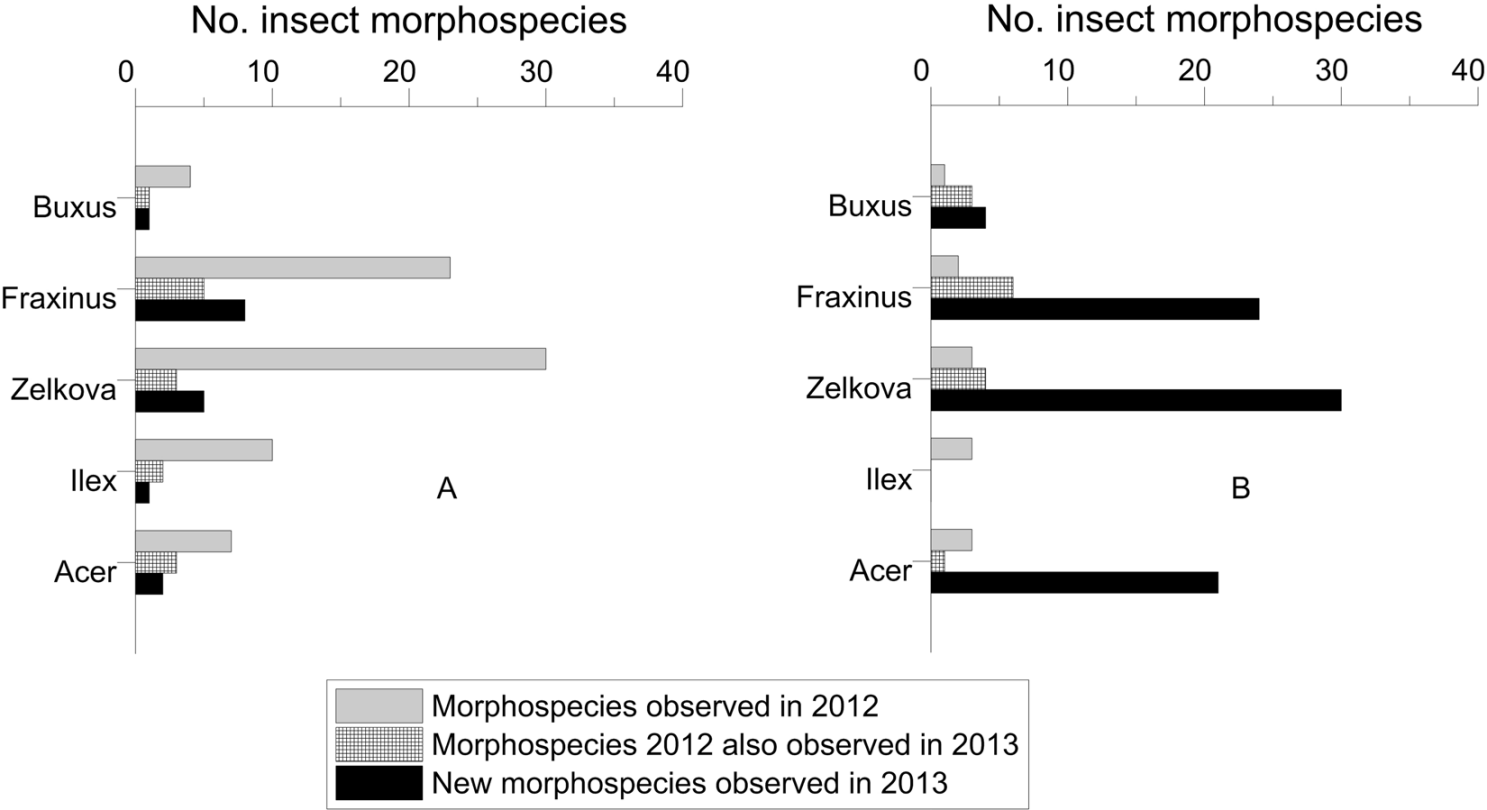
Number of insect morphospecies (including identified and unidentified species) found on the five tree species in Fuyang (left) and Beijing (right) in 2012 only, in 2012 and 2013, and in 2013 only.

**Figure 2:**
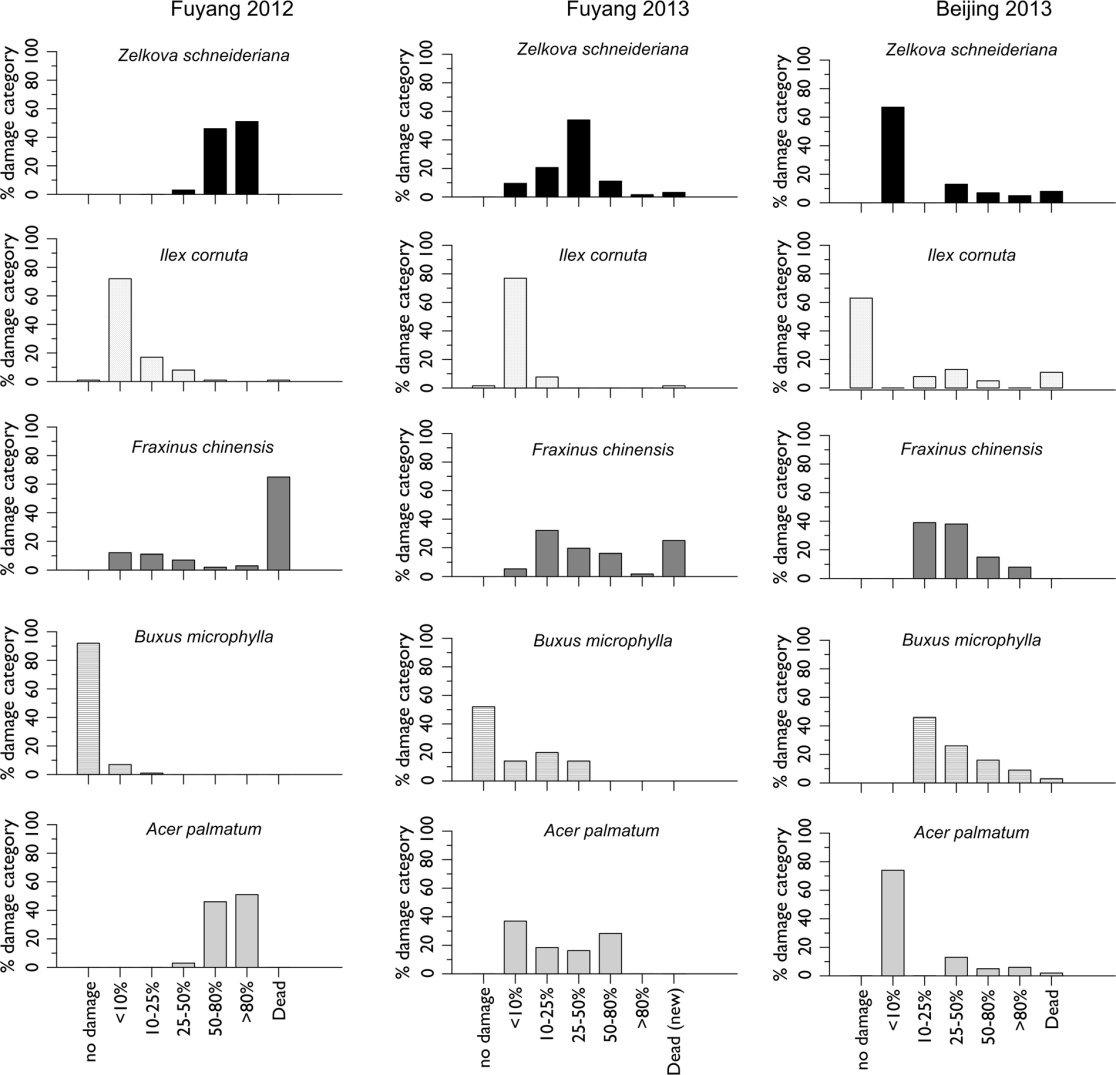
Comparative damage level on the five tree species in Fuyang in 2012 and 2013, and in Beijing in 2013. In Fuyang, the *F. chinensis* plants that died in 2012 were replaced by new seedlings in early 2013.

At both sites, many more insect species were found on the deciduous trees, *Z. schneideriana* and *F. chinensis*, than on the two species with persistent foliage, *B. microphylla* and *I. cornuta*, whereas *A. palmatum* was highly colonised in Beijing but less so in Fuyang (Fig. 1). The general foliar damage levels showed less consistent patterns with variable damage rates among hosts, sites and years (Fig. 2).

Some remarkable damages were observed, with all *B. microphylla* trees at both sites were infested by the box tree moth, *Cydalima perspectalis* (Walker). Seedlings of *Ilex* were infested by an unidentified scale species at Fuyang (65% of the seedlings in 2012 and 18% in 2013, respectively) but not in Beijing. In 2012, about 65% of the *F. chinensis* seedlings in Fuyang died. The removal of dead plants revealed that the roots of ca. half of them had been damaged by chafer larvae of the genus *Holotrichia* spp, already known to have affected sentinel European seedlings previously planted at the same site^19^. However, the other half of the dead seedlings did not show any damaged roots, suggesting that mortality may be due to an unidentified pathogen, but no further analysis was carried out to prove it.

Insect species richness and damage levels increased with time at the two sites, with more insects and damage recorded in the second year of the experiment in Beijing (28 morphospecies × hosts found in 2012 and 95 morphospecies in 2013). However, the rate of accumulation of new insect species was significantly lower at Fuyang during the second year compared to Beijing (Fig. 1). No insects were found on 50 additional plants, 10 of each species, immediately inspected after having been purchased in October 2013.

Among the 92 combinations of insect species, host and region with insects identified to species level from the sentinel nurseries, 89.1% of the insect-host associations had not been found in a general, *a priori* literature survey of pests of the five hosts in China. The *a priori* survey identified 69 insect pests, including many abundant and damaging species (Supplementary Information, Table S3). From a literature survey carried out *a posteriori*, it appeared that all insects identified to species level were known from China but 79.6% of the plant-insect interaction records were not found.

We found that 9.0% of the 210 insect records were considered to present a high likelihood of introduction, 7.6% a moderate likelihood and 83.3% a low likelihood. The latter category comprised mainly insect species that are found only on foliage of broadleaved hosts whereas these hosts are only exported in winter without foliage. In broadleaved hosts, the high likelihood category was represented by insects overwintering in or on stem or branches.

## Discussion

The plant health protection benefits of CRA over PRA have been discussed by several authors^3,11^. For example, considering the huge increase of *Buxus* spp. imports from China to Europe in recent years^25^, a commodity risk assessment would have noted the risk of importing the box tree moth, which is presently devastating ornamental and natural box trees in most European countries and the Caucasus^26,27^. Instead, PRAs were carried out only when the moth had already entered Europe and had become invasive. A CRA based on the literature only would possibly have selected this species as one of the potential pests present on this pathway, but our experiments clearly showed its high occurrence in two different regions and strong negative impacts on plant fitness. Indeed, 48% and 100% of the *B. microphylla* trees were damaged by the moth in 2013 in Fuyang and Beijing, respectively.

Most pest species identified in our experiments are not listed in international or national literature surveys on the pests of the five host plants. A few of the missing host-pest associations were later found in retrospective pest-specific literature surveys, in case of polyphagous pests species, but this is not the way a literature search is made in the framework of a CRA^14,15^. Some of the pests found on the sentinel plants and not in the literature appear to be inconspicuous and could be imported on plants for planting and become major threat to European biodiversity and economy. Examples include insects on *I. cornuta* and *B. microphylla* that are shipped with their leaves, but also insects of broadleaved host trees that feed and/or undergo diapause in or on stems and branches. For these reasons, we recommend the use of sentinel nurseries as part of future CRAs for live plants.

Despite their elevated success in detecting likely insect pests, our sentinel nurseries did not reveal all insect pests related to these trees in China. In fact, over 80% of the insect pests found in the literature surveys were not discovered at the nurseries. Therefore, a sentinel nursery approach should not be seen as a replacement for literature reviews in CRA, but as complementary to them. Additionally, replicating sentinel nurseries is also critical to their utility. The overlap between the two nurseries was very low, which is not surprising given the distance between the two nurseries and the different environments in which they were established. More nurseries in other regions would probably have revealed other pests. Therefore, it is important to place sentinel nurseries in the same regions and environments as the exporting nurseries.

Our data showed that many additional insect pests were found in the second year of the study, particularly in Beijing, where few were found in the first months after planting. This is partly due to the fact that the pest may have needed some time to reach the sites and infest the plants. This reason may also explain why the site in Beijing, set up in an agricultural, peri-urban area, took longer to be infested than the site in Fuyang, situated at the edge of a forest. The delay in infestation may also be due to the chemical treatments applied to the plants before they were purchased. The few plants purchased at exporting nurseries in 2013 were free from insect pests. Plants are normally treated before export to minimise the occurrence of pests. Thus, the exported plants would be much less infested by pests than our sentinel plants. While phytosanitary treatments can aid in the prevention of infested stock being transported, the practical issues with the application of treatments to large consignments of plants may lead to an unequal degree of treatment being applied across the consignment. Therefore, a sentinel nursery with untreated plants would reveal the potential pests carried by exported plants, and if used in conjunction with pest surveys in plant nurseries, would provide a comprehensive list of potential pests on the host plants. Wild plants, or plants used in public gardens could be inspected as well but can sometimes be inaccessible to inspections and, in the case of public gardens, are often treated with pesticides.

The vast majority of the insects were found on leaves. This is not surprising since leaf feeders are usually the most conspicuous insect pests. For the three broadleaved species, which are usually exported to Europe without leaves in winter, leaf-feeders probably represent a low risk. However, some of them will overwinter inconspicuously as eggs on twigs or branches. We found few insects in the winter, including eggs (Supplementary Information, Tables S1, S2), but these are probably the most critical ones and those for which treatments are most needed. More generally, the survey for inconspicuous insect stages in or on the branches would probably require changes in our survey methods, with increasing time spent on each tree. Few xylophagous insects were found, as expected with very small trees. The Fuyang sentinel nursery has been left untouched since the end of the experiment and opportunistic observations made in 2016–2017, i.e. four and five years after plantation, revealed the recruitment of several xylophagous beetles, among which Cerambycidae in the genera *Batocera* and *Anoplophora* (J. Fan and A. Roques, unpublished observations). However, at that time, the plants were too tall for export.

Protocols to identify exotic pests potentially carried by imported plants for planting in the framework of a CRA should include the combination of traditional literature surveys (including the national literature of the exporting country) with surveys of sentinel nurseries. These sentinel nurseries should:

- Be established and monitored for at least two years since relatively few organisms were found in the first season.
- Be set up in regions and environments where the plants are grown and stored before export to reflect the importance capturing observed variation in species pools across regions. Ideally, untreated sentinel plantations should be established and managed by national plant protection organisations in the vicinity of exporting nurseries; exported plants awaiting shipment should not be stored outside of monitored regions without careful protection against pest infestation.
- Be inspected in the season of exportation, with focus on tissues that are actually exported. For example, broadleaved trees that are exported in winter without leaves should have their stems, branches and roots carefully examined in winter. Evergreen trees should have all the plant parts examined throughout the year.
- Insects should also be identified using DNA barcodes, so as to build up an improved library of the potential pests, taking into account that some groups are better represented in the public genetic databases than others.

## Methods

### Establishment of the sentinel nurseries

Two sentinel nurseries about 1200 km apart were established in April 2012 in China. The first was situated in an experimental farm at Yamen village, Nanfaxin town, surrounded by agricultural fields, about 30 km northeast of Beijing (40.1541 N; 116.6021 E; 40 m a.s.l.). The second was established at Fuyang (30.0033 N; 119.7997 E; 110 m a.s.l.), about 40 km southwest of Hangzhou (Zhejiang province, Eastern China), in a small farm completely surrounded by a mixed conifer and broadleaved forest. The region of Beijing is characterised by a temperate, humid continental climate and Fuyang by a humid subtropical climate.

Five woody plant species were selected from the live plants commonly exported from China to Europe at the beginning of the experiment: *A. palmatum, I. cornuta, B. microphylla, F. chinensis* and *Z. schneideriana*. The two nurseries were established using plants bought in the same exporting nursery just before plantation. Since their purchase, the plants were not protected against local pests by any means. Five blocks of 20 plants were planted for each species, i.e. 100 plants per species, or 500 plants per site. No plant was taller than 80 cm, to correspond to typical exported plants. The distance between plants was 50 cm and between blocks 1.5 to 2 m.

In 2012 in Fuyang, about 65% of the *F. chinensis* seedlings died from unknown factors, probably pathogens or chafer larvae (*Holotrichia* spp.). These trees were replaced in winter 2012–2013 by new seedlings. In October 2013, ten additional plants of the five species were bought from a nursery that commonly exports plants to Europe, and they were inspected for insects in the same way as the sentinel plants, for comparison with our untreated plants.

### Inspection for insects and identifications

The two nurseries were surveyed every two weeks from April to October, in 2012 and 2013 (i.e. two seasons of observations), and once a month during the rest of the year. Insect surveys were carried by visual observations and by beating branches over a beating tray. Insect species that may be transported on plants and that are putatively plant pests (i.e. not predators or other organisms that are likely to be on the plant by chance) were collected and preserved for identification. Insects found as adult only that may be pests at a larval stage but do not feed on the plant at the adult stage (e.g. Lepidoptera, Diptera, Hymenoptera) were not considered. Adult insects suspected to feed on plants (e.g. Coleoptera, Hemiptera) were collected, put in 95% alcohol for tentative identification and DNA barcoding. Insect larvae were placed partly in alcohol for barcoding and partly reared to adults in sleeved cages or cages in the laboratory.

Adult insects that could not be morphologically identified by the authors were sent to taxonomists. However, in many cases, a morphological identification could not be obtained and the unidentified species were assigned to morphospecies (defined here as a group of individuals that we recognised as probably belonging to a same species based on morphological characteristics). Similarly, immature stages could only rarely be identified and were also assigned to morphospecies. For 65 morphospecies, adults or immature stages were subjected to molecular analyses using the mtDNA barcode gene in order to compare them with existing barcode datasets, following the methodology described in detail in Roques *et al*.^19^.

General damage levels, including damage by insects but also pathogens, were assessed at the end of the season, in late October 2012 and 2013 in Fuyang and in early October 2013 in Beijing. No assessment was made in Beijing in 2012. Foliar damage was measured by estimating the percentage of damaged leaves on each tree (0%, <10%, 10–25%, 25–50%, 50–80%, >80%, dead). Dead or dying seedlings or branches were cut and examined in the laboratory to assess whether the dying was caused by insects.

### Literature surveys

For the five plant species included in this study, an *a priori* literature survey was carried out before the field assessments (i.e. completed in 2011), using CAB Direct^28^ and Crop Protection Compendium^29^, two classical electronic information tools used to perform PRA and CRA. General Chinese references in entomology and plant protection, such as^30–32^ were also consulted. Lists of the main insect pests reported from the five plant species in China were then compiled, as it would be done for a CRA. Pests known from closely-related congeneric plant species were also noted. At the end of the project, the lists were compared with those recorded from the sentinel nursery. Finally, all pests identified at species level in the sentinel nursery were included an *a posteriori* literature survey to find their known distribution in China and Europe, and whether they were known from the plant on which they were observed. The survey was mainly done by searching Google and Google Scholar with the tree species and the insect species name introduced simultaneously, as well as general entomology books. However, this survey cannot be considered as exhaustive because not all the possible literature sources were used and, thus, some insect-host records may have been missed.

### Risk assessments

Each morphospecies found on a sentinel plant was assessed for its likelihood of being exported with plants, taking into account the period of presence on the plant and its coincidence with the period of export (e.g. winter for deciduous trees), the conspicuousness of the organism or damage. Insects were classified as low likelihood when they were unlikely to be transported because the insect is usually not on the tree when it is exported. For example, this is the case for all species whose larvae feed on foliage of the three broadleaved trees in summer, but eggs are not present on trees in winter, when the trees are traded. They were classified as moderately likely when they can be exported because at least one stage can be found on the plant at the export time and stage, but this stage is obvious with naked eyes in regular inspections; The likelihood was classified as high when at least one stage can be found on the plant at the export time and stage, and the organism is inconspicuous through inspections. Since, for many morphospecies, we did not know the exact biology, in particular whether other developmental stages can be present on the plant and on which part of the plant, the assessment was usually made for the observed stage. However, in the few cases where we had the evidence that other stages can be found on these particular plants, we took this information into account in the assessment.

## Electronic supplementary material

Supplementary information
