# Supplementary material for: Sentinel nurseries to assess the phytosanitary risks from insect pests on importations of live plants
Source: Sci Rep. 2018 Jul 25;8:11217. doi: 10.1038/s41598-018-29551-y (PMC6060114; doi:10.1038/s41598-018-29551-y)
Supplement: Supplementary file 1 — Supplementary information [file 41598_2018_29551_MOESM1_ESM.docx]

Sentinel nurseries to assess the phytosanitary risks from insect pests on importations of live plants

Marc Kenis, Hongmei Li, Jian-ting Fan, Beatrice Courtial, Marie-Anne Auger-Rozenberg, Annie Yart, René Eschen and Alain Roques

Supplementary information

| **Table S1.** Details of the insect species found during surveys at the sentinel nursery in Beijing with results of the *a priori* and *a posteriori* literature surveys, and results of the assessment on likelihood of transport to Europe | | | | | | | | | | |
| --- | --- | --- | --- | --- | --- | --- | --- | --- | --- | --- |
| **Order** | Species | Development stage observed | Identification | Year of occurrence | Number of occurrences | Period of occurrence | Part of plant attacked | A priori literature survey | A posteriori literature survey | Likelihood of transport to Europe |
|  |  | E: Egg;  L: Larva A: Adult | M: Morphol. G: Genetic |  | No of trees x no. of times the species was observed |  |  | Y: Yes N: No () genus only | Y: Yes  N:NO | see text for score |
| **BUXUS** |  |  |  |  |  |  |  |  |  |  |
| **Coleoptera** | *Plagiodera versicolora* (Laicharting) (Chrysomelidae) | A | M | 2013 | 4 | June-Oct. | Leaves | N | N | 1 |
| **Hemiptera** | Aphidae | A | M | 2012 | 3 | June | Leaves |  |  | 3 |
| **Hemiptera** | Berytidae | A | M | 2013 | 1 | Aug. | Leaves |  |  | 2 |
| **Hemiptera** | Cicadellidae | A | M | 2012-13 | 10 | July-Oct. | twigs |  |  | 2 |
| **Hemiptera** | Lygaeidae | A | M | 2013 | 1 | Aug. | twigs |  |  | 2 |
| **Hemiptera** | Membracidae | A | M | 2013 | 3 | Sept.-Oct. | twigs |  |  | 2 |
| **Hemiptera** | *Halyomorpha picus* (Fabricius) (Pentatomidae) | A | M | 2012-13 | 2 | July-Sept. | twigs | N | N | 2 |
| **Lepidoptera** | *Cydalima perspectalis* (Walker) (Crambidae) | L | M&G | 2012-13 | 50 | May-Oct. | leaves and twigs | Y | Y | 3 |
| **FRAXINUS** |  |  |  |  |  |  |  |  |  |  |
| **Coleoptera** | *Pterolophia angusta* (Bates, 1873) (Cerambycidae) | A | M | 2013 | 1 | Aug. | Trunk | N | N | 1 |
| **Coleoptera** | *Protaetia brevitarsis* Lewis (Cetoniidae) | A | M | 2013 | 2 | Aug. | Trunk | Y | Y | 1 |
| **Coleoptera** | *Plagiodera versicolora* (Laicharting) (Chrysomelidae) | A | M | 2013 | 5 | May-Oct. | Leaves | N | N | 1 |
| **Coleoptera** | *Pyrrhalta sp.* (Chrysomelidae) | A | M | 2012-13 | 3 | June-July | Leaves | (N) |  | 1 |
| **Coleoptera** | *Henosepilachna vigintioctomaculata* (Motschulsky) (Coccinellidae) | A | G | 2013 | 1 | Sept. | Leaves | N | Y | 1 |
| **Coleoptera** | Curculionidae | A | M | 2013 | 2 | July-Aug. | twigs |  |  | 1 |
| **Coleoptera** | *Maladera* sp. (Melolonthidae) | A | M | 2013 | 1 | Aug. | Trunk | (N) |  | 1 |
| **Hemiptera** | Riptortus pedestris (Fabricius) (Alydidae) | A | M | 2012-13 | ? | July-Sept. | twigs | N | N |  |
| **Hemiptera** | *Prociphilus fraxinifolii* (Riley) (Aphidae) | L\A | M&G | 2013 | 60 | May-Aug. | Leaves | Y | Y | 1 |
| **Hemiptera** | Aphidae | A | M | 2012 | 2 | July | Leaves |  |  | 1 |
| **Hemiptera** | Berytidae | A | M | 2013 | 1 | June | Leaves |  |  | 1 |
| **Hemiptera** | Meimuna mongolica (Cicadidae) | A | M | 2013 | 1 | Aug. | twigs | N | N | 1 |
| **Hemiptera** | Cryptotympana pustulata (Cicadidae) | A | M | 2013 | 3 | Sept.-Oct. | twigs | N | N | 1 |
| **Hemiptera** | Derbidae | A | M | 2013 | 2 | June-Aug. | twigs |  |  | 1 |
| **Hemiptera** | Dictyopharidae | A | M | 2012 | 1 | Aug. | Leaves |  |  | 1 |
| **Hemiptera** | Lycorma delicatula (Fulgoridae) | A | M | 2012-13 | 45 | May-Nov. | twigs | Y | Y | 1 |
| **Hemiptera** | *Dolycoris baccarum* Linnaeus (Pentatomidae) | A | M | 2013 | 5 | May-Oct. | twigs | N | N | 1 |
| **Hemiptera** | *Halyomorpha picus* (Fabricius) (Pentatomidae) | A | M | 2012-13 | 24 | June-July | twigs | N | N | 1 |
| **Hemiptera** | *Erthesina fullo* (Thunberg) (Pentatomidae) | L | M | 2013 | 2 | Aug. | twigs | N | N | 1 |
| **Lepidoptera** | *Hyphantria cunea* (Drury) (Arctiidae) | L | M&G | 2013 | 30 | Sept.-Oct. | Leaves | Y | Y | 1 |
| **Lepidoptera** | *Ascotis selenaria* Sehiffermüller et Denis (Geometridae) | L | G/M | 2013 | 2 | July-Aug. | leaves | N | N | 1 |
| **Lepidoptera** | Geometridae | L | M | 2013 | 3 | June-Aug. | Leaves |  |  | 1 |
| **Lepidoptera** | Geometridae | L | M | 2013 | 2 | July-Aug. | Leaves |  |  | 1 |
| **Lepidoptera** | Geometridae | L | M | 2013 | 1 | May | Leaves |  |  | 1 |
| **Lepidoptera** | Geometridae | L | M | 2013 | 1 | Sept. | Leaves |  |  | 1 |
| **Lepidoptera** | *Parasa sinica* Moore (Limacodidae) | L | G | 2012-13 | 9 | July-Nov. | Leaves | N | N | 1 |
| **Lepidoptera** | *Euproctis similis* (Füssli) (Lymantriidae) | L | M/G | 2013 | 1 | July | Leaves | N | N | 1 |
| **Lepidoptera** | *Hypenodes humidalis* Doubleday (Noctuidae) | L | G | 2013 | 1 | May | Leaves | N | N | 1 |
| **Lepidoptera** | Pyralidae | L | M | 2013 | 2 | July-Aug. | Leaves |  |  | 1 |
| **Lepidoptera** | *Psilogramma increta* (Walker) (Sphingidae) | L | G | 2012-13 | 2 | July-Sept. | Leaves | N | Y | 1 |
| **Lepidoptera** | Tortricidae | L | M | 2013 | 2 | July-Aug. | Leaves |  |  | 1 |
| **Others** | Unknown eggs | E |  | 2013 | Many | Winter | Branches |  |  | 3 |
| **ZELKOVA** |  |  |  |  |  |  |  |  |  |  |
| **Coleoptera** | *Tomapoderus ruficollis* Fabricius (Attelabidae) | A | M | 2012-13 | 25 | July-Oct. | Leaves | N | Y | 1 |
| **Coleoptera** | Bostrichidae | A | M | 2013 | 5 | April-July | Trunk |  |  | 1 |
| **Coleoptera** | Buprestidae | A/E | M | 2013 | Many | May (L) Winter (E) | Trunk |  |  | 3 |
| **Coleoptera** | Cerambycidae | A | M | 2013 | 1 | June | Trunk |  |  | 1 |
| **Coleoptera** | Cerambycidae | A | M | 2013 | 2 | July | Trunk |  |  | 1 |
| **Coleoptera** | *Plagiodera versicolora* (Laicharting) (Chrysomelidae) | A | M | 2012-13 | 6 | May-Oct. | Leaves | N | N | 1 |
| **Coleoptera** | *Pyrrhalta sp.(Chrysomelidae)* | A | M | 2013 | 1 | June | Leaves | (N) |  | 1 |
| **Coleoptera** | Chrysomelidae | A | M | 2013 | 1 | Oct. | Leaves |  |  | 1 |
| **Coleoptera** | Chrysomelidae | A | M | 2013 | 1 | June | Leaves |  |  | 1 |
| **Coleoptera** | *Henosepilachna vigintioctomaculata* (Motschulsky) (Coccinellidae) | A&L | M | 2013 | 17 | July-Sept. | Leaves | N | N | 1 |
| **Coleoptera** | Curculionidae | A | M | 2013 | 1 | July | twigs |  |  | 1 |
| **Coleoptera** | *Maladera* sp. (Melolonthidae) | A | M | 2013 | 1 | July | Trunk | (Y) |  | 1 |
| **Coleoptera** | *Anomala corpulenta* Motschulsky (Rutelidae) | A | M | 2013 | 1 | July | Trunk | N | N | 1 |
| **Coleoptera** | *Popillia quadriguttata*(Rutelidae) | A | M | 2013 | 2 | July | Trunk | N | N | 1 |
| **Hemiptera** | Aphidae | A | M | 2012 | 34 | June | Leaves |  |  | 1 |
| **Hemiptera** | Berytidae | A | M | 2013 | 5 | June-Sept. | Leaves |  |  | 1 |
| **Hemiptera** | *Cicadella viridis* Linnaeus (Cicadellidae) | A | M | 2012-13 | 38 | May-Oct. | twigs | (N) |  | 1 |
| **Hemiptera** | Derbidae | A | M | 2013 | 1 | April-July | twigs |  |  | 1 |
| **Hemiptera** | Lygaeidae | A | M | 2013 | 2 | July-Aug. | twigs |  |  | 1 |
| **Hemiptera** | Lygaeidae | A | M | 2012 | 1 | June | twigs |  |  | 1 |
| **Hemiptera** | Membracidae | A | M | 2013 | 2 | Sept.Oct. | twigs |  |  | 1 |
| **Hemiptera** | *Dolycoris baccarum* Linnaeus (Pentatomidae) | A | M | 2013 | 2 | May-Aug. | twigs | N | N | 1 |
| **Hemiptera** | *Halyomorpha picus* Fabricius (Pentatomidae) | A | M | 2012-13 | 14 | June-Aug. | twigs | N | N | 1 |
| **Hemiptera** | *Erthesina fullo* (Thunberg) (Pentatomidae) | A | M | 2012 | 1 | Oct. | twigs | Y | Y | 1 |
| **Lepidoptera** | *Chionarctia nivea* (Ménétriés) (Arctiidae) | L | M&G | 2013 | 1 | Aug | Leaves | N | N | 1 |
| **Lepidoptera** | *Chionodes mongolica* Piskunov (Gelechiidae) | L | G | 2013 |  | ? | Leaves | N | N | 1 |
| **Lepidoptera** | *Ascotis selenaria* Schiffermüller et Denis (Geometridae) | L | M&G | 2013 | 1 | July | Leaves | N | N | 1 |
| **Lepidoptera** | Geometridae | L | M | 2013 | 1 | Aug. | Leaves |  |  | 1 |
| **Lepidoptera** | Geometridae | L | M | 2013 | 8 | Jun-Aug | Leaves |  |  | 1 |
| **Lepidoptera** | *Euproctis sp.* (Lymantriidae) | L | M&G | 2013 | 2 | July-Sept. | Leaves | (Y) |  | 1 |
| **Lepidoptera** | *Orgyia* sp. (Lymantriidae) | L | M | 2013 | 3 | Aug. | Leaves | (N) |  | 1 |
| **Lepidoptera** | *Acronicta rumicis* (L.) (Noctuidae) | L | G | 2013 |  | ? | Leaves | N | N | 1 |
| **Lepidoptera** | *Rhodophaea formosa* (Haworth) (Pyralidae) | L | M | 2013 | 6 | June-July | Leaves | N | N | 1 |
| **Lepidoptera** | *Scythris sinensis (Felder et Rogenhofer) (Scythrididae)* | L | M | 2013 | 3 | July-Aug. | Leaves | N | N | 1 |
| **Lepidoptera** | *Choristoneura longicellana* (Walsingham) (Tortricidae) | L | G | 2013 |  | ? | Leaves | N | N | 1 |
| **Lepidoptera** | Tortricidae | L | M | 2013 | 4 | July-Sept. | Leaves |  |  | 1 |
| **Lepidoptera** | Tortricidae | L | M | 2013 | 1 | May | Leaves |  |  | 1 |
| **ILEX** |  |  |  |  |  |  |  |  |  |  |
| **Hemiptera** | Aphidae | L | M | 2012 | 12 | June | Leaves |  |  | 3 |
| **Lepidoptera** | *Parasa sinica* Moore (Limacodidae) | L | M | 2012 | 1 | June | Leaves | N | N | 3 |
| **Lepidoptera** | Limacodidae | L | M | 2012 | 1 | June | Leaves |  |  | 3 |
| **ACER** |  |  |  |  |  |  |  |  |  |  |
| **Coleoptera** | *Plagiodera versicolora* Laicharting (Chrysomelidae) | A | M | 2013 | 12 | May-Oct. | Leaves | N | N | 1 |
| **Coleoptera** | *Pyrrhalta sp* (Chrysomelidae) | A | M | 2013 | 1 | June | Leaves | (N) |  | 1 |
| **Coleoptera** | Chrysomelidae | A | M | 2013 | 4 | May-Sept. | Leaves |  |  | 1 |
| **Coleoptera** | *Henosepilachna vigintioctomaculata* Motschulsky | A | M | 2013 | 1 | Aug. | Leaves | N | N | 1 |
| **Coleoptera** | *Xylinophorus mongolicus* Faust (Curculionidae) | A | M | 2013 | 10 | Aug. | twigs | N | N | 1 |
| **Coleoptera** | *Sympiezomias sp.* (Curculionidae) | A | M | 2013 | 5 | June.July | twigs | (N) |  | 1 |
| **Coleoptera** | Curculionidae | A | M | 2013 | 1 | July | twigs |  |  | 1 |
| **Coleoptera** | *Maladera* sp. (Melolonthidae) | A | M | 2013 | 1 | Aug. | Trunk | (N) |  | 1 |
| **Coleoptera** | *Anomala corpulenta* Motschulsky (Rutelidae) | A | M | 2013 | 1 | July | Trunk | N | N | 1 |
| **Hemiptera** | Berytidae | A | M | 2013 | 2 | Aug.-Oct. | Leaves |  |  | 1 |
| **Hemiptera** | *Cicadella viridis* Linnaeus (Cicadellidae) | A | M | 2013 | 20 | July-Oct. | twigs | N | Y | 1 |
| **Hemiptera** | Derbidae | A | M | 2013 | 2 | Sept. | twigs |  |  | 1 |
| **Hemiptera** | Lygaeidae | A | M | 2013 | 10 | June-Nov. | twigs |  |  | 1 |
| **Hemiptera** | Lygaeidae | A | M | 2012 | 1 | June | twigs |  |  | 1 |
| **Hemiptera** | Membracidae | A | M | 2013 | 17 | Sept.-Oct. | twigs |  |  | 1 |
| **Hemiptera** | *Eurydema gebleri* (Kolenati) Pentatomidae | A | M | 2012 | 1 | June | twigs | N | N | 1 |
| **Hemiptera** | *Halyomorpha picus* (Fabricius) (Pentatomidae) | A | M | 2013 | 1 | Aug. | twigs | N | N | 1 |
| **Lepidoptera** | Chionarctia nivea (Ménétriés) (Arctiidae) | L | G | 2013 |  |  | Leaves | N | N | 1 |
| **Lepidoptera** | Ascotis selenaria Sehiffermüller et Denis (Geometridae) | L | M&G | 2013 | 1 | Sept. | Leaves | N | N | 1 |
| **Lepidoptera** | Geometridae | L | M | 2013 | 2 | June-Sept | Leaves |  |  | 1 |
| **Lepidoptera** | Geometridae | L | M | 2013 | 3 | June-Aug. | Leaves |  |  | 1 |
| **Lepidoptera** | *Cnidocampa flavescens* (Walker) (Limacodidae) | L | M | 2012-13 | 2 | July-Aug. | Leaves | N | Y | 2 |
| **Lepidoptera** | *Parasa sinica* Moore (Limacodidae) | L | M | 2012 | 1 | July | Leaves | N | N | 1 |
| **Lepidoptera** | Helicoverpa armigera (Hübner) (Noctuidae) | L | M&G | 2013 | 1 | Aug | Leaves | N | N | 1 |
| **Lepidoptera** | *Scythris sinensis* (Felder et Rogenhofer) ( Scythrididae | L | G | 2013 |  | ? | Leaves | N | N | 1 |

| **Table S2.** Details of the insect species found during surveys at the sentinel nursery in Fuyang, with results of the *a priori* and *a posteriori* literature surveys, and results of the assessment on likelihood of transport to Europe | | | | | | | | | | |
| --- | --- | --- | --- | --- | --- | --- | --- | --- | --- | --- |
| **Order** | Species | Development stage observed | Identification | Year of occurrence | Number of occurrences | Period of occurrence | Part of plant attacked | A priori literature survey | A posteriori literature survey | Likelihood of transport to Europe |
|  |  | E: Egg;  L: Larva A: Adult | M: Morphol. G: Genetic |  | No of trees x no. of times the species was observed |  |  | Y: Yes N: No () genus only | Y: Yes  N:NO | see text for score |
| **BUXUS** |  |  |  |  |  |  |  |  |  |  |
| **Coleoptera** | Galeruca sp. 1 (Chrysomelidae) | A | M | 2012 | 5 | April to July | leaves | (N) |  | 1 |
| **Coleoptera** | Chrysomelidae sp. 1 | A | M | 2012 | 1 | April | leaves |  |  | 1 |
| **Hemiptera** | Pentatomidae sp. 2 | A | M | 2012 | 3 | October | leaves |  |  | 1 |
| **Hemiptera** | Pentatomidae sp. 5 | L | M | 2012 | 4 | October | leaves |  |  | 1 |
| **Hymenoptera** | Tenthredinidae sp 3 | L | M & G | 2013 | 5 | July | leaves |  |  | 3 |
| **Lepidoptera** | Cydalima perspectalis (Walker) (Crambidae) | L, A, E | M & G | 2012-13 | many | April to October | leaves | Y | Y | 3 |
| **FRAXINUS** |  |  |  |  |  |  |  |  |  |  |
| **Coleoptera** | Asthates episcopalis Chevrolat (Cerambycidae) | A | M | 2012 | 1 | June | Leaves | N | N | 1 |
| **Coleoptera** | Lema sp. 1 (Chrysomelidae) | A | M | 2012 | 6 | June | Leaves | (N) |  | 1 |
| **Coleoptera** | Cryptocephalus sp. 3 (Chrsomelidae) | A | M | 2012 | 3 | August | Leaves | (N) |  | 1 |
| **Coleoptera** | Cryptocephalus sp.1 (Chrysomelidae) | A | M | 2012 | 4 | June | Leaves | (N) |  | 1 |
| **Coleoptera** | Smaragdina nigrifrons (Hope) (Chrysomelidae) | A | M | 2012 | 12 | June- July | Leaves | N | N | 1 |
| **Coleoptera** | nr Adoretus (Rutelidae) | L | G | 2013 | 2 | October | Roots |  |  | 2 |
| **Coleoptera** | Holotrichia sp. 1 (Melolonthidae) | L | M | 2012 | 3 | November | Roots | (Y) |  | 2 |
| **Coleoptera** | Holotrichia trichophora Fairm. (Melolonthidae) | A | M | 2012 | 2 | November | Leaves | Y | Y | 1 |
| **Hemiptera** | Cicadellidae sp. 2 | A | M | 2012 | 2 | November | Leaves |  |  | 1 |
| **Hemiptera** | Cicadellidae sp. 1 | A | M | 2012 | 2 | May | Leaves |  |  | 1 |
| **Hemiptera** | Lygaeidae sp. 1 | A | M | 2012 | 1 | August | Leaves |  |  | 1 |
| **Hemiptera** | Lygaeidae sp. 2 | A | M | 2012 | 1 | August | Leaves |  |  | 1 |
| **Hemiptera** | Pentatomidae sp. 4 | A | M | 2012 | 3 | October | Leaves |  |  | 1 |
| **Hemiptera** | Pentatomidae sp. 6 | A | M | 2012 | 3 | October | Leaves |  |  | 1 |
| **Hymenoptera** | Tenthredinidae sp 2 | L, A | M | 2012 | 6 | July | Leaves |  |  | 1 |
| **Hymenoptera** | Tenthredinidae sp. 1 | L | M | 2013 | 6 | June | Leaves |  |  | 1 |
| **Lepidoptera** | Cossus nr. insularis (Cossidae) | P | M | 2013 | 3 | October | Stem | N | N | 3 |
| **Lepidoptera** | Ganisa similis Moore (Eupterotidae) | P | G | 2012- 13 | 8 | June to October | Leaves | N | N | 1 |
| **Lepidoptera** | Geometridae sp 3 nr. Charissa | L | G | 2012 | 1 | June- July | Leaves |  |  | 1 |
| **Lepidoptera** | Problepsis nr. albidior Warren (Geometridae) | L | G | 2012 | 5 | July | Leaves | N | N | 1 |
| **Lepidoptera** | Problepsis sp. 2 (Geometridae) | L | G | 2013 | 1 | June | Leaves | (N) |  | 1 |
| **Lepidoptera** | Limacodidae sp. 1 | L | G | 2012 | 6 | November | Leaves |  |  | 1 |
| **Lepidoptera** | Limacodidae sp. 2 | L | G | 2012 | 4 | November | Leaves |  |  | 1 |
| **Lepidoptera** | Euproctis piperita Oberthür or E. fimbriata (T.P.Lucas) (Lymantriidae) | L, P | G | 2012- 13 | 12 | October | Leaves | N | N | 1 |
| **Lepidoptera** | Euproctis similis (Fuessly) (Lymantriidae) | L | G | 2013 | 6 | October | Leaves | N | N | 1 |
| **Lepidoptera** | Pangrapta lunulata Stertz (Noctuidae) | L | G | 2013 | 4 | September | Leaves | N | N | 1 |
| **Lepidoptera** | Acronicta rumicis Linnaeus (Noctuidae) | L | G | 2012- 13 | 10 | August to October | Leaves | N | N | 1 |
| **Lepidoptera** | Spodoptera litura Fabricius (Noctuidae) | E, L | G | 2012- 13 | 8 | October | Leaves | N | N | 2 |
| **Lepidoptera** | Palpita quadristigmalis Bremer or P. nigropunctalis (Guenée) (Pyralidae) | L | G | 2013 | 8 | June to October | Leaves | N | Y | 1 |
| **Lepidoptera** | Psychidae sp. 2 | P | G | 2013 | 2 | October | Leaves |  |  | 1 |
| **Lepidoptera** | Psychidae sp. 3 | L | G | 2012 | 8 | November | Leaves |  |  | 1 |
| **Lepidoptera** | Acherontia styx medusa Moore (Sphingidae) | L | G | 2012 | 1 | August | Leaves | N | Y | 1 |
| **Lepidoptera** | Dolbina inexacta Walker (Sphingidae) | P | G | 2012- 13 | 4 | June to October | Leaves | N | Y | 1 |
| **Lepidoptera** | Psilogramma increta (Walker) (Sphingidae) | P | G | 2012 | 3 | November | Leaves | N | Y | 1 |
| **Lepidoptera** | Lepidoptera eggs isolated | E | G | 2012 | 1 | November | Leaves |  |  | 2 |
| **Orthoptera** | Orthoptera sp. 3 | A | M | 2012 | 1 | May | Leaves |  |  | 1 |
| **ZELKOVA** |  |  |  |  |  |  |  |  |  |  |
| **Coleoptera** | Anoplophora chinensis Forster (Cerambycidae) | A | M & G | 2012 | 1 | July | Leaves, Stem | N | Y | 1 t0 3 |
| **Coleoptera** | Cerambycidae sp. 1 | L | M | 2012 | 1 | November | Stem |  |  | 2 |
| **Coleoptera** | Paraglenea fortunei (Saunders) (Cerambycidae) | L | M & G | 2012 | 1 | November | Stem | N | N | 3 |
| **Coleoptera** | Bruchinae sp. 1 (Chrysomelidae) | A | M | 2012 | 1 | June | Leaves |  |  | 1 |
| **Coleoptera** | Galeruca sp. 1 (Chrysomelidae) | A | M | 2012 | 7 | June- July | Leaves | (N) |  | 1 |
| **Coleoptera** | Lema sp. 1 (Chrysomelidae) | A | M | 2012 | 5 | June- July | Leaves | (N) |  | 1 |
| **Coleoptera** | Mimastra limbata Baly (Chrysomelidae) | A | M & G | 2013 | 3 | June | Leaves | N | N | 1 |
| **Coleoptera** | Chrysomelidae sp. 1 | A | M | 2012 | 3 | May | Leaves |  |  | 1 |
| **Coleoptera** | Cryptocephalus sp. 3 (Chrysomelidae) | A | M | 2012 | 5 | June- July | Leaves | (N) |  | 1 |
| **Coleoptera** | Chrysomelidae sp. 2 | A | M | 2012 | 5 | June- July | Leaves |  |  | 1 |
| **Coleoptera** | Smaragdina nigrifrons (Hope) (Chrysomelidae) | A | M | 2012 | 9 | June- July | Leaves | N | N | 1 |
| **Coleoptera** | Curculionidae sp. 1 | A | M | 2012 | 1 | June- July | Leaves |  |  | 1 |
| **Coleoptera** | Holotrichia diomphalia Bates (Melolonthidae) | A | M | 2012 | 1 | June | Leaves | N | N | 1 |
| **Coleoptera** | Holotrichia titanus Reitter (Melolonthidae) | L | M | 2012 | 4 | November | Roots | N | N | 2 |
| **Coleoptera** | Holotrichia sp. 2 (Melolonthidae) | L | M | 2012 | 4 | November | Roots | (N) |  | 2 |
| **Coleoptera** | Anisoplia sp. (Rutelidae) | A | M | 2012 | 12 | June- July | Leaves | (N) |  | 1 |
| **Coleoptera** | Mimela splendens (Gyllenhal) (Rutelidae) | A | M | 2012 | 15 | June- July | Leaves | N | N | 1 |
| **Coleoptera** | Popilia sp. 1 (Rutelidae) | A | M | 2012 | 2 | June- July | Leaves | (N) |  | 1 |
| **Hemiptera** | Aphididae sp. 1 | L, A | M | 2012 | 14 | June to November | Leaves |  |  | 1 |
| **Hemiptera** | Cicadellidae sp. 1 | A | M | 2012 | 1 | July | Leaves |  |  | 1 |
| **Hemiptera** | Coreidae sp. 1 | A | M | 2012 | 2 | October | Leaves |  |  | 1 |
| **Hemiptera** | Pentatomidae sp. 1 | L, A | M | 2012 | 1 | July | Leaves |  |  | 1 |
| **Hemiptera** | Pentatomidae sp. 3 | A | M | 2012 | 2 | July | Leaves |  |  | 1 |
| **Hemiptera** | Pentatomidae sp. 7 | A | M | 2012 | 1 | November | Leaves |  |  | 1 |
| **Hemiptera** | Pentatomidae sp. 8 | A | M | 2012 | 3 | November | Leaves |  |  | 1 |
| **Hymenoptera** | Tenthredinidae nr Caliroa/ Empria | L | G | 2013 | 5 | July to November | Leaves |  |  | 1 |
| **Hymenoptera** | Tenthredinidae sp 2 | L, A | G | 2012 | 6 | July | Leaves |  |  | 1 |
| **Lepidoptera** | Arctiidae | L | G | 2012 | 2 | October | Leaves |  |  | 1 |
| **Lepidoptera** | Geometridae sp 2 nr. Paradromulia/ Jankowskia | L | G | 2013 | 2 | September- October | Leaves |  |  | 1 |
| **Lepidoptera** | Geometridae sp. 1 nr Jankowskia | L | G | 2013 | 3 | September- October | Leaves |  |  | 1 |
| **Lepidoptera** | Calliteara grotei Moore (Lymantriidae) | L | G | 2012 | 2 | October | Leaves | N | N | 1 |
| **Lepidoptera** | Euproctis piperita Oberthür or E. fimbriata (T.P.Lucas)(lymantriidae) | L, P | G | 2012- 13 | 6 | September- October | Leaves | N | N | 1 |
| **Lepidoptera** | Acronicta rumicis Linnaeus (Noctuidae) | L | G | 2012- 13 | 8 | August- October | Leaves | N | N | 1 |
| **Lepidoptera** | Pangrapta nr. vasava (Butler) (Noctuidae) | L | G | 2013 | 2 | June | Leaves | N | N | 1 |
| **Lepidoptera** | Spodoptera litura Fabricius (Noctuidae) | E, L | G | 2012- 13 | 9 | October | Leaves | N | N | 2 |
| **Lepidoptera** | Vanessa indica Herbst (Nymphalidae) | L | G | 2012 | 1 | July | Leaves | Y | Y | 1 |
| **Others** | Orthoptera sp. 1 | A | M | 2012 | 1 | November | Leaves |  |  | 1 |
| **Others** | Orthoptera sp. 2 | L | M | 2012 | 1 | May | Leaves |  |  | 1 |
| **ILEX** |  |  |  |  |  |  |  |  |  |  |
| **Coleoptera** | Stenygrinum quadrinotatum Bates (Cerambycidae) | A | M | 2012 | 1 | June | Leaves | N | N | 1 |
| **Coleoptera** | Cryptocephalus sp. 2 (Chrysomelidae) | A | M | 2012 | 3 | June- July | Leaves | (N) |  | 1 |
| **Coleoptera** | Galeruca sp. 1 (Chrysomelidae) | A | M | 2012 | 5 | June- July | Leaves | (N) |  | 1 |
| **Coleoptera** | Galeruca sp. 2 (Chrysomelidae) | A | M | 2012 | 4 | June- July | Leaves | (N) |  | 1 |
| **Coleoptera** | Smaragdina nigrifrons (Hope) (Chrysomelidae) | A | M | 2012 | 9 | June- July | Leaves | N | N | 1 |
| **Hemiptera** | Ceroplastes nr. rubens Maskell (Coccidae) | L, A | M | 2012-13 | 12 | July to November | Stem and twigs | Y | Y | 3 |
| **Hemiptera** | Pentatomidae sp. 7 | A | M | 2012 | 2 | November | Leaves |  |  | 1 |
| **Lepidoptera** | Euproctis piperita Oberthür or E. fimbriata (T.P.Lucas)(lymantriidae) | L, P | G | 2012-13 | 8 | October | Leaves | N | N | 3 |
| **Lepidoptera** | Capusa nr senilis Walker(Geometridae) | L | G | 2012 | 1 | July | Leaves | N | N | 3 |
| **Lepidoptera** | Eurychoria sp. (Geometridae) | L | G | 2013 | 1 | October | Leaves | (N) |  | 3 |
| **Lepidoptera** | nr Pteroma (Psychidae) | L | G | 2012 | 10 | July to November | Leaves |  |  | 3 |
| **Lepidoptera** | Archips sp. (Tortricidae) | L | G | 2012 | 1 | November | Leaves | (Y) |  | 3 |
| **Lepidoptera** | Zygaenidae sp. 1 | L | G | 2012 | 1 | November | Leaves |  |  | 3 |
| **ACER** |  |  |  |  |  |  |  |  |  |  |
| **Coleoptera** | Eumolpinae sp. 1 (Chrysomelidae) | A | M | 2012 | 2 | May- June | Leaves |  |  | 1 |
| **Coleoptera** | Elateridae sp. 1 | A | M | 2012 | 1 | May- June | Leaves |  |  | 1 |
| **Coleoptera** | Melolontha sp. 1 (Melolonthidae) | L | M | 2012 | 2 | May- June | Roots | (N) |  | 2 |
| **Coleoptera** | Anisoplia sp. (Rutelidae) | A | M | 2012 | 8 | May- June | Leaves | (N) |  | 1 |
| **Coleoptera** | Mimela splendens (Gyllenhal) (Rutelidae) | A | M | 2012 | 15 | May- June | Leaves | N | N | 1 |
| **Hemiptera** | Pentatomidae sp. 1 | L, A | M | 2012 | 2 | June | Leaves |  |  | 1 |
| **Lepidoptera** | Geometridae sp 4 | L | G | 2013 | 4 | September to November | Leaves |  |  | 1 |
| **Lepidoptera** | Euproctis piperita Oberthür or E. fimbriata (T.P.Lucas)(lymantriidae) | L, P | G | 2012-13 | 6 | October | Leaves | N | N | 1 |
| **Lepidoptera** | Euproctis similis (Fuessly)(Lymantriidae) | L | G | 2013 | 3 | October | Leaves | N | N | 1 |
| **Lepidoptera** | Acronicta rumicis Linnaeus (Noctuidae) | L | G | 2012-13 | 8 | August to October | Leaves | N | N | 1 |
| **Lepidoptera** | Spodoptera litura Fabricius (Noctuidae) | E, L | G | 2012-13 | 8 | September to November | Leaves | N | N | 2 |
| **Lepidoptera** | Pempelia formosa (Haworth) (Pyralidae) | L | G | 2012 | 1 | July | Leaves | N | N | 1 |

| **Table S3.** Insect species associated with the tree species in an a priori literature search | | |
| --- | --- | --- |
|  | Order: Family | Source |
| **Buxus microphylla** |  |  |
| *Abraxa* *(Calospilos)* *suspecta* (Warren)* | Lep.: Geometridae | CABI |
| *Abraxas miranda* Butler | Lep.: Geometridae | Nat |
| *Chrysomphalus* *(Aspidiotus* = *Lindigaspis*) rossi (Maskell)^#^ | Hem.: Diaspididae | CABI |
| *Cydalima perspectalis* (Walker) | Lep.: Crambidae | CABI, Nat |
| *Homona magnanima* Diakonoff | lep.: Tortricidae | Nat |
| *Icerya purchasi* Maskell | Hem.: Margarodidae | Nat |
| *Lymantria dispar* (L.) | Lep.: Lymantriidae | Nat |
| *Parlagena buxi* (Takahashi) | Hem.: Diaspididae | CABI, Nat |
| *Pinnaspis buxi* (Bouché)* | Hem.: Diaspididae | CABI |
| *Ricania sublimbata* Jacobi^#^ | Hem.: Ricaniidae | CABI |
| *Toxoptera aurantii* (Boyer de Fonscolombe) | Hem.: Aphididae | Nat |
| *Unaspis euonymi Comstock）* | Hem.: Diaspididae | Nat |
| *Yponomeuta* sp.* | Lep.: Yponomeutidae | CABI |
|  |  |  |
| **Zelkovia schneideriana** |  |  |
| *Adoretus tenuimacutatus* Waterhouse | Col.: Rutelidae | Nat |
| *Agrotis ipsilon* (Hufnagel) (= *A. ypsilon* (Rottemberg)) | Lep.: Noctuidae | Nat |
| *Batocera horsfieldi* (Hope) | Col.: Curculionidae | Nat |
| *Cyphochilus insulanus* Moser | Col. Scarabaeidae | Nat |
| *Eriogyna pyretorurn* Westwood | Lep.: Saturniidae | Nat |
| *Erthesina fullo* (Thunberg) | Hem.: Pentatomidae | Nat |
| *Euproctis* (=*Porthesia*) *similis* Füssli) | Lep: Lymantriidae | Nat |
| *Hypomeces squamosus* (Fabricius) | Col.: Curculionidae | Nat |
| *Lamiomimus gottschei* Kolbe | Col.: Cerambycidae | Nat |
| *Maladera* (=*Autoserica*) *japonica* (Motschulsky) | Col.: Scarabaeidae | Nat |
| *Maladera orientalis* Motschklsky | Col. Scarabaeidae | Nat |
| *Phassus sinifer sinensis* Moore | Lep.: Hepialidae | Nat |
| *Planociampa antipala* Prout* | lep.: Geometridae | CABI |
| *Rhizoecus hibisci* Kawai & Takagi* | Hem.: Pseudoccidae | CABI |
| *Sagra femorata* (Drury) | Col.:: Chrysomelidae | Nat |
| *Tetraneura zelkovisucta* Zhang (= *Paracolopha morrisoni* (Baker) | Hem.: Aphididae | Nat, CABI |
| *Theretra oldelandiae* (Fabricius) | Lep.: Sphingidae | Nat |
| *Vanessa indica* (Hbst) | Lep: Nymphalidae | Nat |
| *Zeuzera pyrina* (L) | Lep.: Cossidae | Nat |
|  |  |  |
| **Ilex cornuta** |  |  |
| *Archips seminubilus* (Meyrick)* | Lep. Tortricidae | CABI |
| *Ceroplastes rubens* Maskell | Hem.: Coccidae | Nat |
| *Homona coffearia* (Nietner)* | Lep. Tortricidae | CABI |
|  |  |  |
| **Fraxinus chinensis** |  |  |
| *Agrilus planipennis* Fairmaire | Col.: Buprestidae | CABI, Nat |
| *Amphimallon solstitialis* *mesasiaticus* Medvedev* | Col.: Scarabaeidae | CABI |
| *Batocera horsfieldi* (Hope) | Col.: Curculionidae | CABI, Nat |
| *Drosicha corpulenta* (Kuwana) | Hem.: Margarodidae) | Nat |
| *Dyscerus cribripennis* Matsumura & Kono | Col.: Curculionidae | Nat |
| *Ericerus pela* (Chavannes) | Hem.: Coccidae | CABI |
| *Holotrichia trichophora* (Fairmaire) | Col.: Scarabaeidae | Nat |
| *Hylesinus eos* Spessivtsev* | Col.: Curculionidae | CABI, Nat |
| *Hyphantria cunea* (Drury) | Lep.: Arctiidae | CABI |
| *Lepyrus japonicus* Roelofs | Col.: Curculionidae | Nat |
| *Longitarsus bimaculatus* Baly* | Col.: Chrysomelidae | CABI |
| *Lycorma delicatula* (White) | Hem.: Fulgoridae | Nat |
| *Lymantria dispar* (L) | Lep.: Lymantriidae | CABI |
| *Mesosa myops* (Dalman) | Col.: Cerambycidae | Nat |
| *metabolus flavescens*  Brenske^#^ | Col.: Scarabaeidae | CABI |
| *Orthosia incerta* Hfn | Lep.: Noctuidae | CABI |
| *Parthenolecanium corni* (Bouché) | Hem.: Coccidae | Nat |
| *Phassus excrescens* (Butler)* | Lep.: Hepialidae | CABI |
| *Phenacoccus fraxinus* Tang | Hem.: Pseudococcidae | CABI, Nat |
| *Phenacoccus perillustris* Borchsenius | Hem.: Pseudococcidae | CABI |
| *Prociphilus fraxini* (F.) | Hem.: Aphididae | CABI |
| *Prociphilus fraxini* F. | Hem.: Aphididae | Nat |
| *Protaetia* (=*Potosia*) *brevitarsis* (Lewis) | Col.: Scarabaeidae | Nat |
| *Pseudococcus comstocki* (Kuwana) | Hem.: Pseudococcidae | CABI |
| *Rhopalosiphum nymphaeae* (L.) | Hem.: Aphididae | Nat |
| *Sinoxylon japonicus* Lesne | Col.: Bostrychidae | Nat |
| *Temnaspis nankinea* (Pic) | Col.: Chrysomelidae | CABI, Nat |
|  |  |  |
| **Acer palmatum** |  |  |
| *Anacampsis populella* (Clerck)* | Lep.:Gelechiidae | CABI |
| *Anoplophora chinensis* (Forster) | Col. Cerambycidae | CABI, Nat |
| *Anoplophora glabripennis* (Motschulsky)* | Col. Cerambycidae | CABI |
| *Caloptilia* sp.* | Lep.: Gracillariidae | CABI |
| *Dasychira pudibunda* (L.) | Lep: Lymantriidae | Nat |
| *Parasa* (= *Latoia*) *pastoralis* Butler | Lep Limacodidae | Nat |
| *Popillia japonica* Edward Newman | Col.: Scarabaeidae | CABI, Nat |

*Associated with a closely-related congeneric species

#Associated with the tree genus but not precisely with the tree species

§ CABI: Reference 28; Nat: National Chinese literature, references 30-32 and others
